# Cognitive Remediation in Schizophrenia: Current Status and Future Perspectives

**DOI:** 10.1155/2013/156084

**Published:** 2013-12-17

**Authors:** Stefano Barlati, Giacomo Deste, Luca De Peri, Cassandra Ariu, Antonio Vita

**Affiliations:** ^1^Department of Mental Health, Spedali Civili Hospital, Piazzale Spedali Civili 1, 25123 Brescia, Italy; ^2^Department of Clinical and Experimental Sciences, University of Brescia, Viale Europa 11, 25123 Brescia, Italy

## Abstract

*Objectives*. This study is aimed to review the current scientific literature on cognitive remediation in schizophrenia. In particular, the main structured protocols of cognitive remediation developed for schizophrenia are presented and the main results reported in recent meta-analyses are summarized. Possible benefits of cognitive remediation in the early course of schizophrenia and in subjects at risk for psychosis are also discussed. *Methods*. Electronic search of the relevant studies which appeared in the PubMed database until April 2013 has been performed and all the meta-analyses and review articles on cognitive remediation in schizophrenia have been also taken into account. *Results*. Numerous intervention programs have been designed, applied, and evaluated, with the objective of improving cognition and social functioning in schizophrenia. Several quantitative reviews have established that cognitive remediation is effective in reducing cognitive deficits and in improving functional outcome of the disorder. Furthermore, the studies available support the usefulness of cognitive remediation when applied in the early course of schizophrenia and even in subjects at risk of the disease. *Conclusions*. Cognitive remediation is a promising approach to improve real-world functioning in schizophrenia and should be considered a key strategy for early intervention in the psychoses.

## 1. Introduction 

Impairments in a wide range of cognitive abilities have been consistently reported in individuals with schizophrenia [1]. In the recent past, the Measurement and Treatment Research to Improve Cognition in Schizophrenia (MATRICS) project has identified seven distinct cognitive domains that are impaired in patients with schizophrenia: speed of processing, attention/vigilance, working memory, verbal and visual learning, reasoning and problem solving, and social cognition [2]. Moreover, in the third meeting of the Cognitive Neuroscience Treatment Research to Improve Cognition in Schizophrenia (CNTRICS) project, it was agreed that six areas or cognitive domains suffered impairment in schizophrenia: perception, working memory, attention, executive functions, long-term memory, and social cognition [3]. Social cognitive deficits include impairments in facial affect recognition, in perceiving and interpreting social cues, theory of mind (ToM), and the ability to make appropriate causal attributions for events [4]. Both neurocognitive and social cognitive deficits are thought to underlie the severe functional disabilities associated with schizophrenia, and several studies have shown that cognitive deficits are related to social deficits and poorer outcomes in different functional domains [5–7]. The influence of cognition on functional outcomes may occur through its influence on functional capacity, the ability to perform critical everyday living skills [8]. Functional capacity has been found to be quite strongly related to cognitive performance and may actually be considered as an intermediate step between neurocognition and everyday functioning [9]. With this more detailed knowledge of the role and meaning of cognitive deficits in schizophrenia, improvement in cognitive functions has become a relevant target in the care and clinical management of the illness [10]. Although pharmacological treatment has been shown to be effective in reducing psychotic, particularly positive, symptoms cognitive impairment has mostly been found to be poorly affected by such treatments [11]. Major initiatives are under way to find new nonpharmacological treatments for cognitive impairment in schizophrenia with the aim of improving also patients' functional outcomes. Newer psychosocial interventions and cognitive rehabilitation treatment approaches are framed in a positive light that are grounded in a *recovery* rather than *deficit *model [12]. This new emphasis is based on the factors associated with improved quality of life, such as the ability to enjoy social and familial interactions, advance in educational endeavors, and performing well at work. The underlying theoretical framework comes from a developmental neuroscience perspective, which supports the idea that the brain is capable of changes and development throughout the lifespan. Most cognitive interventions are based, in principle, on the large literature supporting the concept of brain plasticity and neurogenesis [13]. Cognitive science assumes that skills development can occur at any age and can help advance or restore the brain's capacity for improving cognitive or social performance [14]. Learning in a properly stimulating environment can help the patient to capitalize on brain malleability and improve functioning [15]. In this context, cognitive remediation attempts to improve and/or restore cognitive functioning using a range of approaches.

In this comprehensive review we aimed to increase the knowledge and understanding of the principles and methodology of cognitive remediation interventions for schizophrenia and highlight the evidence of effectiveness of such interventions deriving from the current scientific literature. First, we present the general principles and features of cognitive remediation and describe the main structured protocols developed for schizophrenia. Then we review the main results reported in recent meta-analyses of the efficacy of remediation interventions in experimental conditions as well as its effectiveness “in the real world.” We also examined the existing evidence of possible benefits deriving from cognitive remediation in the early course of schizophrenia and in subjects “at risk” of psychosis. Finally, we investigated the potential neurobiological correlates of the effects of cognitive remediation in schizophrenic patients. The data, acquired on the efficacy, the neurobiological mechanisms of the effects of cognitive remediation, and its usefulness in the early course of schizophrenia and reported for the first time in a single systematic review, could contribute both to improving our knowledge on the possibility to interfere with the trajectory of brain pathology of schizophrenia and to designing new treatments for the disease that combine effectiveness and personalization.

## 2. Cognitive Remediation in Schizophrenia: Definition, Methods, and Techniques

Cognitive rehabilitation has been defined as “the therapeutic process of increasing or improving an individual's capacity to process and use incoming information so as to allow increased functioning in everyday life. This includes methods to train and restore cognitive function and compensatory techniques” [16]. Cognitive remediation for schizophrenia has been recently defined as “a behavioural training based intervention that aims to improve cognitive processes (attention, memory, executive function, social cognition or metacognition) with the goal of durability and generalisation” (Cognitive Remediation Experts Workshop (CREW), Florence, April 2010). Cognitive remediation strategies can be distinguished into two main models: “compensatory” and “restorative” [17]. The “compensatory” treatments try to eliminate or to bypass the specific cognitive deficit, using the subject's residual cognitive abilities and/or the environmental resources. Indeed, the manipulation of the environment is a compensatory technique acting and operating changes in the environment in order to influence and facilitate the cognitive functions, for example, by simplifying the patient's tasks [18]. On the other hand, the “restorative” methods are based on knowledge deriving from neurosciences, in particular neuronal plasticity, and have the objective to correct a specific deficit trying to repair the specific underlying compromised function using the capacity of the brain to develop and repair itself throughout the whole life [10, 14]. Restorative remediation strategies utilize two different approaches: bottom-up or top-down. Bottom-up approaches start with remediation of basic neurocognitive skills, such as attention, and advance to more complex skills, such as problem solving. In contrast, top-down approaches use more complex skills with the aim of improving single and specific neurocognitive domains [19]. Thus, some restorative techniques take into account the use of drill and practice exercises, in order to restore cognitive functions and, possibly, improve neuronal plasticity, while others are based on the implementation of new strategies and tend to favour the generalization in different contexts through the execution of different tasks that involve the use of similar strategies [10, 20]. Cognitive remediation utilizes several learning strategies, including errorless learning, scaffolding, massed practice, positive reinforcement, and information processing strategies [20]. Errorless learning appears to be effective because it avoids the implicit encoding of errors which cannot then be differentiated from correct information by explicit recall. Scaffolding is similar to errorless learning in ensuring a high degree of success for the learner and minimising errors, by carefully regulating the complexity of material to be learnt. The learner is encouraged to use previously established areas of competence, whilst help is provided with new aspects of learning. Massed practice consists in the exercise of a repeated task (at least 2-3 times per week) in order to encourage the retention and application of the skills developed. Information processing strategies include verbalization, information reduction, breaking and simplifying the task into smaller steps, providing written prompts, chunking, self-monitoring, mnemonic strategies, categorization, organization, and planning. These strategies are applied differently and to varying degrees in different methods of cognitive remediation, depending on whether they are primarily based on repeated execution of specific tasks or on the implementation of new strategies. Cognitive remediation can be delivered as a package that provides a standard set of exercises, or it may be personalized to only target deficits identified in the single individual. Some cognitive remediation programs focus on a specific cognitive domain (e.g., working memory or facial affect recognition), whereas others are broad-based, incorporating multiple domains. It is clearly possible that all cognitive remediation strategies are complementary and synergic and that the potentiation of specific target functions may favour the development of new compensatory strategies of problem solving, which could be applied and influence the patient's daily life [10, 20, 21]. Several factors have influence of a positive treatment response for cognitive remediation training, such as training of the therapist, motivation of the patient, intensity and type of training, and baseline cognitive resources [10].

### 2.1. Cognitive Remediation in Schizophrenia: The Main Structured Protocols

In recent decades, a number of cognitive remediation techniques, computerized and noncomputerized, designed for individual or group settings, have been developed and adopted in multimodal treatment approaches in schizophrenia. The main structured protocols of cognitive training for schizophrenia are described in Table 1 [22].

## 3. Materials and Methods

### 3.1. Search Strategy

Electronic searches were performed in the PubMed database combining the following search terms: “schizophrenia,” “cognitive remediation,” “cognitive training,” “neurocognitive enhancement,” “cognitive rehabilitation,” “functional outcome,” “meta-analysis,” “neurobiological correlates,” “first episode psychosis,” “early schizophrenia,” and “at risk psychosis.” Detailed combinations of the above search terms are available from the authors on request. Two of the authors (Stefano Barlati, Giacomo Deste) independently reviewed the database in order to avoid errors in the selection of articles. In addition, the reference lists of the included articles were carefully hand-searched to further identify other studies of possible interest.

### 3.2. Selection Criteria

All the studies, meta-analyses, and review articles on cognitive remediation in schizophrenia published until April 2013 have been included. Studies were included according to the following criteria: (a) being an original paper published in a peer-reviewed journal and (b) having performed experiments using a cognitive remediation technique in schizophrenia. Studies on psychological, psychosocial, or psychoeducational interventions only, without any cognitive remediation approach or technique, were not considered.

## 4. Results

### 4.1. Cognitive Remediation in Schizophrenia: Evidence from Meta-Analyses

To date, various published meta-analyses support the efficacy of cognitive remediation for improving cognitive outcomes targeted by these interventions. More than ten years ago, Pilling et al. [23], in a first review based on few studies, reported that cognitive remediation had no benefit on attention, verbal memory, visual memory, planning, cognitive flexibility, or mental state and concluded that cognitive remediation did not appear to confer reliable benefits for patients with schizophrenia and could not be recommended for clinical practice. In more recent years, several quantitative reviews have well established that cognitive remediation is effective in reducing cognitive deficits and in improving functional outcome with long-term benefits in schizophrenia [24–31].  Table 2 summarizes the main results of meta-analytic studies on cognitive remediation in schizophrenia. In one of these quantitative reviews, McGurk et al. [27] showed that cognitive rehabilitation is associated not only with an improvement of cognitive functions, but also with a slightly less significant improvement of psychosocial functioning and symptoms of schizophrenia. The most recent meta-analysis of the available controlled studies of cognitive remediation in schizophrenia performed by Wykes et al. [31] showed a moderate improvement in overall cognitive performance, with some durability of the effects, as shown in follow-up studies (ES = 0.43). Moreover, there was a significant small-to-medium effect on functional outcomes at both posttreatment and follow-up assessment (ES = 0.37). The results of these two meta-analyses highlighted that the most significant effects on social functioning can be demonstrated when cognitive training is administered together with other psychosocial rehabilitation programs, and when a strategy coaching approach based on learning strategies is adopted. The investigations performed by Roder et al. [28, 29] indicate that integrated psychological therapy (IPT) is an effective rehabilitation approach for schizophrenia that is robust across a wide range of patient characteristics and treatment conditions. The authors highlight that the cognitive and social subprograms of IPT may work in a synergistic manner, thereby enhancing durability of therapy effects and improving functional recovery. In a meta-analysis performed by Grynszpan et al. [24], computer-assisted cognitive remediation (CACR) techniques, which enable selective treatment of different cognitive domains, have been shown to improve a wide range of cognitive domains and social cognition in schizophrenia. A recent meta-analysis performed by Kurtz and Richardson [26], specifically on social cognitive interventions, stressed the greatest effect of treatments on facial affect recognition (FAR), with a moderate-to-large effect size for affect identification and a large effect size for affect discrimination. Authors also reported a moderate effect size for ToM and a large impact on measures of observer-rated community and institutional functioning.

### 4.2. Cognitive Remediation in Schizophrenia: Efficacy in the Early Course of Schizophrenia and in Subjects “at Risk” of Psychosis

First study on cognitive remediation in adolescents in the early course of psychosis was conducted by Ueland and Rund [32]. This randomized controlled study demonstrated that a cognitive remediation program might have beneficial effects for some specific aspects of cognition and possibly an indirect effect on measures of functional outcome in this group of patients. The same research group performed a second study investigating the long-term effects of the cognitive remediation program for adolescents with early onset psychosis [33]. A significant overall improvement for eight of ten cognitive and three of four outcome measures was found. Wykes et al. [34] tried a different approach to cognitive remediation, testing with a randomized controlled design the effects of cognitive remediation therapy (CRT) versus usual treatment in subjects with a recent diagnosis of early-onset schizophrenia (onset prior to the age of 19 and duration of illness of less than 3 years) [20]. Although all cognitive tests showed an advantage for the CRT group, the effect was significant only for the Wisconsin Card Sorting Test (WCST). Another research group studied the effects of a comprehensive paradigm of cognitive remediation (cognitive enhancement therapy, CET), investigating the impact of cognitive training on different outcome measures and also on brain morphology in a number of papers [35–37]. The first step on this path was a randomized controlled trial aimed at investigating the effects of CET on social cognition [35], which demonstrated a significant superiority of CET over a nonspecific treatment. A subsequent randomized controlled trial investigated the effects of a two-year treatment with CET [36]. After the first year of treatment, subjects in the cognitive remediation group showed significant and medium-to-large differential improvements in dysfunctional cognitive style, social cognition, social adjustment, and symptomatology as compared with those in the control condition. After two years of treatment, highly significant and large differential effects were observed, again favouring CET, on the composite indexes of cognitive style, social cognition, social adjustment, and symptomatology. A long-term follow-up study was then performed in order to verify the durability of the effects of CET [37]. Results from intent-to-treat analyses indicated that CET effectiveness on functional outcome was broadly maintained one-year posttreatment and that patients receiving CET continued to demonstrate highly significant differential functional benefits, compared with the control group. A recent study aimed to determine the effectiveness of cognitive remediation (Neuropsychological Educational Approach to Remediation, NEAR) as an early intervention in first-episode depressive and psychotic disorders [38]. Patients undergoing NEAR improved significantly more than treatment-as-usual (TAU) patients in attention, working memory, and immediate learning and memory. Similarly, the cognitive remediation group demonstrated greater improvements in psychosocial functioning. Bowie et al. [39] evaluated the effectiveness and transfer to functional competence and everyday functioning of cognitive remediation in early course (within 5 years of first episode) and long-term (more than 15 years of illness) schizophrenia. The early course group had larger improvements in measures of processing speed and executive functions, in adaptive competence, and real-world work skills.

Only three studies analyzing the efficacy of cognitive remediation techniques in the prodromal phase of schizophrenia or in subjects at risk for schizophrenia were identified [40–42]. Rauchensteiner et al. [40] examined the differential effects of Cogpack [43] in prodromal patients, compared with patients with fully manifested schizophrenia. The results indicate that prodromal patients can improve their long-term verbal memory, attention, and concentration after cognitive training. Another study investigated short-term outcomes of CACR in adolescents with psychotic disorders or at high risk of psychosis [41]. The analysis of data revealed significant differences between baseline and followup in executive function and reasoning abilities, with better performances at followup only in the CACR group. A multicentric, prospective, randomised trial with two parallel groups assigned to alternative outpatient interventions was performed to investigate the effects of an integrated psychological intervention (IPI) on the prevention of psychosis in the so-called “early initial prodromal state” (EIPS) [42]. The incidence of and time of conversion to subthreshold psychotic symptoms, psychosis, and schizophrenia/schizophreniform disorder during a 12-month treatment period were significantly lower for patients who received specially designed IPI than for those who were treated with supportive counselling. Furthermore, IPI appeared effective in delaying the onset of psychosis over a 24-month time period in people in an EIPS. Since IPI covered a variety of psychological strategies, the trial design did not allow assessing the relative contribution of each intervention, including cognitive remediation.

### 4.3. Cognitive Remediation in Schizophrenia: Neurobiological Correlates

Cognitive remediation may determine neurobiological changes, which provides evidence of its biological validity. The changes that have been found to occur indicate the activation of brain repair mechanisms [44]. For instance, Vinogradov et al. [45] reported that patients who received cognitive remediation manifested an improvement in their serum levels of brain-derived neurotrophic factor (BDNF). Wykes et al. [46] found that patients treated with the CRT showed an increase in activation in right inferior frontal gyrus, as well as both right and left occipital lobe, as assessed with functional magnetic resonance imaging (*f*MRI), as compared with the control group. In a recent randomized controlled trial, Penadés et al. [47] reported that the brain networks activation pattern significantly changed in patients exposed to the CRT in the sense of normalization, toward the pattern observed in healthy control subjects. Moreover, the CRT group showed an increase in fractional anisotropy index in the anterior part of the genu of the corpus callosum. The authors concluded that the improvement in brain functioning detected after CRT in schizophrenic patients might be based on an increase of the interhemispheric information transfer between the bilateral prefrontal cortices via the corpus callosum.

There are only two published studies on the neurobiological correlates of cognitive remediation in the early course of schizophrenia [13, 48]. Eack et al. [13], in a structural MRI (*s*MRI) study, reported that while patients in the control condition demonstrated progressive loss of gray matter volume in the fusiform and parahippocampal gyrus, patients receiving CET demonstrated gray matter preservation in these areas, and a significant gray matter volume increase in the left amygdala. These differential effects on gray matter changes were significantly related to improved cognitive functions over the two-year followup. In a subsequent study, the same research group found that the higher baseline cortical surface area and gray matter volume broadly predicted social-cognitive response to CET [48].

## 5. Conclusions and Future Directions

The bulk of available data does demonstrate the efficacy of cognitive remediation interventions on cognitive and psychosocial functioning of patients suffering from schizophrenia. These benefits appear to be especially relevant for chronic and severe patients with schizophrenia, for which cognitive remediation interventions have been now widely applied. Common and consistent results emerge from the available studies, allowing some general statements. Improvements have been found in a wide range of outcomes, including cognition, social cognition, independent living skills, and social adjustment. Less pronounced and at best indirect may be the effects on patient's psychopathology. Cognitive remediation programs appear to be more successful if they are embedded in comprehensive rehabilitation programs where the skills training or cognitive remediation exercises are used in combination with psychosocial groups or work rehabilitation programs [27, 31]. Overall, cognitive remediation is most likely to impact functional outcome when individuals are given opportunities to practice the cognitive skills in real-world settings [17]. In order to achieve generalization to daily functioning it is necessary to include cognitive remediation in broader programs in conjunction with other psychosocial interventions [27, 31]. In particular, by integrating cognitive remediation programs, especially with strategy coaching approach, and psychosocial rehabilitation programs, patients' functional outcomes may be significantly enhanced [31]. In this regard, a prospective controlled study performed by our group established the effectiveness of the cognitive subprograms of IPT (IPT-Cog) on neuropsychological and functional outcome variables, compared with a TAU condition, while both groups continued to receive other rehabilitative interventions [49]. In a subsequent study, in the same rehabilitation context, we have demonstrated that, following 24 weeks of treatment, the IPT-Cog and a CACR program had significant, even if modest, effects on psychosocial functioning. These data confirm the effectiveness of different modalities of cognitive remediation for schizophrenia and the potential for generalization to functional outcomes when provided in a more comprehensive rehabilitation context [50].

Treating cognitive deficits may also be considered as a potential tool to prevent or delay the onset of schizophrenia in a primary (e.g., in high risk population) and secondary (e.g., in subjects with recent onset disease) prevention framework [51]. Evidence emerging from the research literature indicates that targeting cognitive impairments in the early course of schizophrenia can result not only in cognitive improvement *per se*, but also in significant functional benefits in such critical domains as social functioning, employment, and role functioning [35, 36, 52]. These analyses also suggest that such therapy may have clinical utility if integrated into treatment programs of young people with schizophrenia within the “critical period” for early intervention, thus offering a possible opportunity to alter the course of the disease. The “protective” role of early effective intervention on the neurobiological and clinical deteriorating course of the disease [53], proposed for treatment with antipsychotics, especially with the 2nd generation compounds [54], may therefore be extended to nonpharmacological approaches, like cognitive remediation. Since cognitive deficits occur before the onset of psychoses [55] and are significantly associated with poor premorbid adjustment and functional outcome in ultrahigh-risk individuals and in the prodromal phase of schizophrenia [56], there is a clear rationale for further research into cognitive remediation in these populations. Given the theoretical and clinical interest of the possible role of treatments for preventing the subsequent conversion to psychosis in subjects with “at risk mental states” [57], and the present debate on the risk-benefit ratio and ethical concerns of exposing young people to antipsychotic treatment, it would be particularly relevant to assess whether nonpharmacological strategies of intervention could demonstrate a similar preventive efficacy. Furthermore, future research should address more systematically the neurobiological effects of cognitive remediation treatment, *per se* or as an integrated treatment with different classes of antipsychotics, in different phases of illness, in particular their potential to reduce or counteract the progressive brain changes known to occur in schizophrenia [58]. Moreover, future research on cognitive training in schizophrenia should try to shed light on many issues, which currently remain open and/or controversial, among which are the specific and unspecific effects of treatment, the active elements of interventions, the mediators and moderators of their effectiveness, the persistence over time and the generalization of improvements, and the role of motivation, that of metacognition and social cognition for treatment outcome [59, 60]. It will also be helpful to understand which patients might benefit from cognitive remediation interventions and identify possible predictors of individual response [61]. In addition, the rules and methodologies regarding the delivery of different interventions should be better fixed: indications, timing and duration, frequency of participation in the program, intensity of the training sessions, and type of education strategies needed. The new theoretical models developed should take into account this complexity, and the information acquired should be used to design treatments that combine effectiveness, efficiency, and personalization, with favourable cost-benefit ratio. Further research should also address the practical applicability of cognitive remediation techniques in routine clinical practice, in order to assess whether their widespread implementation in mental health services may be recommended.

## Figures and Tables

**Table 1:** Structured protocols of cognitive remediation interventions for schizophrenia (modified from Vita et al. [22]).

Cognitive Training	Target	Duration	Setting (individual/ group)	Computer assisted/ Noncomputer assisted	Restorative/ compensatory	Top-down	Bottom-up	Drill and practice	Strategy coaching	Individually tailored
IPT [62]	Cognitive functions, social skills, and problem solving	Sessions of 60 minutes, 2-3 times a week (about 12 months)	Group (6–8)	Noncomputer assisted	Restorative	+	+	+	+	−
INT [63]	Cognitive functions and social cognition	30 biweekly sessions, 90 minutes each	Group (6–8)	Computer assisted sessions and noncomputer-assisted sessions	Restorative	+	+	+	+	−
CRT [64]	Cognitive functions	40 sessions at least 3 times a week, 45–60 minutes each one	Individual	Not computer assisted session	Restorative	+	+	+	+	+
Cogpack* [43]	Cognitive functions	Sessions variable in duration and frequency (starting from 2-3 weeks)	Individual	Computer assisted	Restorative	−	+	+	−	+
CET [65]	Cognitive functions and social cognition	Biweekly sessions (about 90 minutes every week) for 24 months	Group (couples and then groups of 3-4 couples)	Computer-assisted sessions and noncomputer-assisted sessions	Restorative	+	+	+	+	−
NEAR [66]	Cognitive functions and problem solving	Sessions of 60 minutes, twice a week (about 4 months)	Individual/group (3–10)	Computer-assisted sessions and noncomputer-assisted sessions	Restorative	+	−	−	+	+
NET [67]	Cognitive functions and social cognition	Sessions of 45 minutes at least 5 times a week (about 6 months)	Individual/group	Computer-assisted sessions and noncomputer-assisted sessions	Restorative	−	+	+	−	+
CAT [68]	Cognitive functions	Variable (short weekly visits at home, lasting about 30 minutes)	Individual	Noncomputer assisted	Compensatory	−	−	−	−	+
TAR [69]	Social cognition	12 sessions twice a week, 45 minutes for each one	Small groups of two patients and a therapist	Computer-assisted sessions and noncomputer-assisted sessions	Restorative/ compensatory	−	+	+	+	+
SCIT [70]	Social cognition	24 weekly sessions, 50 minutes each (about 6 months)	Group (6–8)	Computer-assisted sessions and noncomputer-assisted group sessions	Restorative	−	+	+	+	−
SCST [71]	Social cognition	12 weekly sessions, 60 minutes each (about 3 months)	Group (6 patients)	Computer-assisted sessions and noncomputer-assisted group sessions	Restorative	−	+	+	+	−
SCET [72]	Social cognition, ToM	36 sessions of 90 minutes, twice a week (about 6 months)	Group	Noncomputer assisted	Restorative	−	+	+	+	−
MCT [73]	Metacognition	8 biweekly sessions of 45–60 minutes (one cycle per month)	Group (3–10)	Noncomputer assisted	Restorative	+	−	−	+	−
SSANIT [74]	Cognitive functions, social cognition, and social skills	NT: biweekly sessions of 1 hour SST: weekly sessions of 2 hours Duration: 6 months	Individual (group)	NT sessions: computer assisted SST sessions: noncomputer assisted	Restorative	+	+	+	+	+

CAT: cognitive adaptation training; CET: cognitive enhancement therapy; CRT: cognitive remediation therapy; INT: integrated neurocognitive therapy; IPT: integrated psychological therapy; MCT: metacognitive training; NEAR: neuropsychological educational approach to remediation; NET: neurocognitive enhancement therapy; NT: neurocognitive training; SCET: social cognition enhancement training; SCIT: social cognition and interaction training; SCST: social cognitive skills training; SSANIT: social skills and neurocognitive individualized training; SST: social skills training; TAR: training of affect recognition; ToM: theory of mind.

*Cogpack is a typical computer-assisted cognitive remediation (CACR) technique.

**Table 2:** Meta-analyses of the efficacy of cognitive remediation in schizophrenia.

Authors	Types of study	Number of included studies (number of patients)	Main investigated areas	Cognitive remediation program	Clinical outcomes (average effect size*)	Neurocognitive outcomes (average effect size*)	Functional outcomes (average effect size*)	Main findings
Kurtz et al. [25]	RCT and laboratory studies	11 (181)	Executive functions (performance on WCTS) Attention Memory	Remediation strategies for improving performance on WCST	Not investigated	Improvement in executive functions: large mean ES (*d* = 0.98) Attention: mixed results Memory: nonconclusive results	Not investigated	Perseverative errors, categories achieved, and conceptual level responses can be improved utilizing extra instructions, repeated practice, or reinforcement

Twamley et al. [30]	RCT	17 (695)	Symptoms, cognitive performance and functioning	Computer assisted and noncomputer assisted, with and without strategy coaching and compensatory strategies	Reduction in symptom severity: small-to-medium ES (*d* = 0.26)	Improvement in neuropsychological performance: small-to-medium ES (*d* = 0.32)	Improvement in everyday functioning: small-to-medium ES (*d* = 0.51)	Both different types of approaches, computer assisted or not, have effective components that hold promise for improving cognitive performance, symptoms, and everyday functioning

Roder et al. [28]	RCT and open studies	30 independent IPT studies (1393)	Symptoms, cognitive performance, and functioning	IPT (a group program that integrates neurocognitive, social cognitive, and psychosocial rehabilitation)	Reduction in symptom severity: moderate ES (*d* = 0.50)	Improvement in neuropsychological performance: moderate ES (*d* = 0.54)	Improvement in psychosocial functioning: moderate ES (*d* = 0.41)	IPT obtained similarly favorable effects across the different outcome domains, assessment formats, settings, and phases of treatment

McGurk et al. [27]	RCT	26 (1151)	Symptoms, cognitive performance, and functioning	Individual versus group setting, computer versus noncomputer assisted, with and without strategy coaching, compensatory strategies, and social cognitive training	Reduction in symptom severity: small ES (*d* = 0.28)	Improvement in cognitive performance: medium ES (*d* = 0.41)	Improvement in psychosocial functioning: small-to-medium ES (*d* = 0.35)	The impact of cognitive remediation on functional outcomes is significantly greater in studies that also provided psychiatric rehabilitation, suggesting that these two treatment approaches may work in a synergistic way

Grynszpan et al. [24]	RCT	16 (805)	Cognitive performance and social cognition	Computer-assisted cognitive remediation (CACR)	Not investigated	Improvement in general cognition: small-to-moderate ES (*d* = 0.38) Improvement in social cognition: moderate ES (*d* = 0.64)	Not investigated	The results support the efficacy of CACR particularly in social cognition. The difficulty in targeting specific domains suggests a “nonspecific” effect of CACR

Wykes et al. [31]	RCT	40 (2104)	Symptoms, cognitive performance, and functioning	Individual versus group setting, computer versus noncomputer assisted, with and without strategy coaching, compensatory strategies, and social cognitive training	Reduction in symptom severity: small ES (*d* = 0.18), but no longer significant at followup	Improvement in global cognitive performance: moderate ES (*d* = 0.45) Improvement in social cognition moderate ES (*d* = 0.65)	Improvement in psychosocial functioning: moderate ES (*d* = 0.42)	Significantly stronger effects on functioning are found when CR is provided together with another psychiatric rehabilitation. A much larger effect is present when a strategic approach is adopted

Roder et al. [29]	RCT and open studies	36 independent IPT studies (1601)	Symptoms, cognitive performance, social cognition, and functioning	IPT	Reduction in symptom severity: moderate ES (*d* = 0.52)	Improvement in neuropsychological performance: moderate ES (*d* = 0.53) Improvement in social cognition: moderate-to-large ES (*d* = 0.70)	Improvement in psychosocial functioning: moderate ES (*d* = 0.42)	The cognitive and social subprograms of IPT may work in a synergistic manner, thereby enhancing the transfer of therapy effects over time and improving functional recovery

Kurtz and Richardson [26]	RCT	19 (692)	Social cognition, symptoms, and community and institutional functioning	Social cognitive training	Reduction in symptoms: moderate-to-large ES (*d* = 0.68) No significant effect on positive and negative symptoms	Improvement in social cognition: (i) moderate-to-large ES on FAR: identification (*d* = 0.71) and discrimination (*d* = 1.01), (ii) small-to-moderate ES on ToM (*d* = 0.46) No significant effect on social cue perception and attributional style	Improvement in psychosocial functioning: moderate-to-large ES (*d* = 0.78)	This is the first meta-analysis on social cognitive training in schizophrenia. Social cognitive training is effective in improving community and institutional functioning

CR: cognitive remediation; ES: effect size (Cohen's *d*); FAR: facial affect recognition; RCT: randomized controlled trials; WCST: Wisconsin Card Sorting Test.

*Effects were categorized as small (*d* < 0.5), moderate-large (*d* = 0.5–0.8), or large (*d* > 0.8 or greater) [75].
